# Correspondence

**Published:** 1884-10

**Authors:** A. S. Heath


### CORRESPONDENCE.

*To the Editor of the Journal of Comparative Medicine and Surgery:*

DEAR SIR—The statement frequently seen circulating in the newspapers that the Holstein, or Jersey, or some other breed was more susceptible to pleuro-pneumonia, foot and mouth disease, or some other contagious malady, is not borne out by the truth as revealed by reliable statistics.

That the Jersey breed of cattle should be more afflicted in the Illinois contagion is accounted for by the simple fact that they have been exposed more than all the other breeds of the country because they have been purchased from every section of the country, and ten times as many animals of this breed have changed hands than all the other breeds combined.

We believe that the State and United States authorities are handling the matter ably and successfully.

Yours truly,

A. S. HEATH.

